# Stimuli are perceived as lasting longer when there is something bright on the screen

**DOI:** 10.3758/s13414-025-03120-8

**Published:** 2025-07-13

**Authors:** Hakan Karsilar, Sebastiaan Mathôt, Hedderik van Rijn

**Affiliations:** Department of Psychology, University of Groningen, Grote Kruisstraat 2/1, 9712TS Groningen, The Netherlands

**Keywords:** Time perception, Brightness, Pupil size, Arousal, Temporal bisection

## Abstract

Perceived time often diverges from physical time. This discrepancy is important given the crucial role of time perception in numerous cognitive processes. A critical question concerning the non-veridicality of timing is whether and how different physical attributes (e.g., size, speed, and numerosity) influence perceived duration. The present study deals specifically with how perceived time depends on stimulus brightness, both of a to-be-timed stimulus and the background on which this stimulus is presented. The results of two experiments show that increased brightness lengthens perceived duration, and, surprisingly, that this is the case both for the stimulus and the background. The finding that stimulus brightness affects time perception is a much needed replication of classic studies; however, the finding that background brightness similarly affects time perception is novel, and suggests that time perception may be biased by low-level visual perception. Additionally, we tested the hypothesis that large pupils (as a result of spontaneous pupil-size fluctuations) are associated with longer perceived durations. This hypothesis was based on the common assumption that arousal affects both pupil size and time perception; however, in contrast to this hypothesis, results show that pupil size has no relation to perceived time. Taken together, our study suggests that time perception is strongly affected by low-level visual input (brightness) but not—or hardly—by pupil-linked arousal.

## Introduction

Our ability to perceive time underlies many different cognitive processes, such as attention, working memory, language, and decision-making (Allman et al., 2014; Buhusi & Meck, 2005; Wittmann, 2013). The capacity to use time-based information has been deemed a “sense” in its own right. Yet time perception is fundamentally unlike other senses, such as vision or hearing, as it lacks a dedicated sensory organ (Ivry & Schlerf, 2008; Lewis & Miall, 2003). The question is therefore: If time perception is indeed a unique sense, then what exactly is being sensed, and what are the rules that govern this sensory modality? (Basgol et al., 2021; Grondin, 2010; Merchant et al., 2013)

A significant amount of research into the nature of time perception has focused on instances where it diverges from veridicality (i.e., when perceived time differs from real time; Grondin, 2010; Matthews & Meck, 2016; Pariyadath & Eagleman, 2007; Ulrich et al., 2006). Humans often overestimate durations of larger objects, faster movements, higher frequencies, louder sounds, higher pitches, and larger quantities (Berglund et al., 1969; Brown, 1995; Droit-Volet, 2013; Eagleman & Pariyadath, 2009; Herbst et al., 2013; Karsilar et al., 2016, 2019; Karsilar et al., 2018; Matthews & Meck, 2016; Ono & Kawahara, 2007; Penton-Voak et al., 1996; Rammsayer & Lima, 1991; Rammsayer & Verner, 2016; Thomas & Cantor, 1976; Xuan et al., 2007). Neurophysiological research has corroborated these discoveries by demonstrating that the brain’s processing of time shares common pathways in the parietal and frontal regions with processing of quantity and size (Bueti & Walsh, 2009; Cai & Connell, 2015; Cantlon et al., 2009; Casasanto & Boroditsky, 2008; Walsh, 2003; Wittmann et al. 2010). Collectively, these studies suggest that perception of time is mostly indirect, derived from physical and statistical features in the environment.

It is challenging to explain the connections between time perception and nontemporal stimulus properties, such as brightness, which is the focus of the present study. However, information-processing models of timing have been notably effective in addressing this issue (Creelman, 1962; Gibbon et al., 1984; Treisman, 1963). Such models hypothesize an internal clock that produces time-representative pulses, where the integration of these pulses code for an interval (Church, 1984; Gibbon, 1977; Malapani & Fairhurst, 2002; Treisman et al., 1990; van Rijn et al., 2014; Wearden, 1999; Wearden, 2003). This clock can run faster or slower depending on the properties of sensory input as well as internal processes, such as arousal (Allan, 1979; Wearden, 2008), attention (Lejeune & Wearden, 2009), and other nontemporal task demands (Buhusi & Meck, 2005; Fortin & Rousseau, 1987, 1998; Wearden, 1991; Zakay & Block, 1997; see also Coull et al., 2011; Meck, 2006). For instance, heightened arousal in response to an intense stimulus, such as a scary photo, is thought to accelerate the production of time-representative pulses, thus resulting in a longer perceived duration (Angrilli et al., 1997; Droit-Volet & Meck, 2007; Gil & Droit-Volet, 2012; Mella et al., 2011; Nather & Bueno, 2011; Sackett et al., 2010; Smith et al., 2011). Conversely, shifting focus away from a task’s temporal aspects is thought to decelerate the production of pulses, thus resulting in a shorter perceived duration (Block & Gruber, 2014; Coull et al., 2004; Fortin, 2003; Tse et al., 2004; Zakay, 1998; Zakay & Block, 1996, 1997). It is important to note that the “internal clock” model of timing has also been criticized for its high degree of flexibility, to the point of being unfalsifiable (Staddon & Higa, 1999). In fact, it is difficult to create a scenario where an effect of nontemporal stimulus properties on temporal behavior would *not* be explained by a combination of arousal and attention-related processes, both of which are general concepts encompassing a wide range of psychological phenomena. Additionally, the idea of an internal clock which accumulates “time-representative pulses” akin to a stop-watch lacks biological plausibility (Salet et al., 2022; see also Bi & Zhou, 2020; Goel & Buonomano, 2014; Raphan et al., 2019 for alternative modeling approaches). Consequently, although the internal-clock model provides a convenient framework through which stimulus properties can be said to affect subjective time, it is more of an abstract account of how timing takes place in natural environments than a realistic one.

Goldstone and Goldfarb (1964a, b) were among the first to demonstrate the interaction between time and other physical dimensions. The researchers presented two consecutive durations using a red-light-emitting diode and found that the participants tended to perceive a duration as longer when the comparison stimulus was brighter than the standard (see also Goldstone et al., 1978). Two decades later, Brigner (1986) replicated these findings by presenting visual stimuli with different light intensities and durations, this time including flickering and nonflickering stimuli. Brigner’s findings revealed that increasing the light intensity caused participants to perceive time intervals as longer. It is important to note that these pioneering studies exploring the relationship between brightness and time perception have, surprisingly, not been replicated or expanded upon using contemporary methodologies. Particularly, it remains unclear whether it is the brightness of the timed stimulus that causes the overestimation of time, or whether it is the overall brightness of visual input—which includes both the timed stimulus and its visual environment—that matters (Matthews, 2011). This distinction is critical, because it has crucial implications for the role of low-level vision in time perception.

Following the theoretical framework of information-processing theory of timing (and see Salet et al., 2022), the clock pulse rate is not only determined by low-level stimulus features, such as brightness, but also by higher-level cognitive and affective processes. Notably, increased arousal is widely thought to accelerate the pulse rate, thus resulting in longer perceived durations (Gil & Droit-Volet, 2012; Lake et al., 2016; Mella et al., 2011; Nather et al., 2011; Van Volkinburg & Balsam, 2014). A key finding in support of this was reported by Suzuki et al. (2016), who measured pupil size in primates, which is known to be an index of arousal (Beatty & Lucero-Wagoner, 2000; Bradley et al., 2008; Mathôt, 2018; Partala & Surakka, 2003). The primates were trained to perform a saccadic eye movement after a specific interval had elapsed. Crucially, the authors found that primates made faster saccades when their pupils were large, suggesting that high (pupil-linked) arousal indeed resulted in longer perceived durations. However, a recent study by Warda et al. (2022) arrived at a different conclusion. Here, the authors measured pupil size while human participants performed a temporal bisection task in which participants reported whether a probe stimulus was presented for a long or a short duration (relative to two reference durations). In contrast to the study by Suzuki et al. (2016), the authors did not find that large pupils were associated with longer perceived durations, which in this paradigm would translate into a response bias towards long reports; rather, they found that large pupils were associated with increased response precision, presumably because pupil dilation is associated with increased motivation to perform the task well. Taken together, there is some evidence suggesting that arousal as indexed by pupil size affects time perception (see also Akdoğan et al., 2016; Kinzuka et al., 2021), but the evidence is mixed and thus warrants further scrutiny.

Our study aims to fill the gap in understanding how perceived duration is affected by both low-level visual features (notably brightness) and arousal. Previous studies have suggested that increased brightness and increased arousal both result in longer perceived duration; however, evidence is mixed and the specific conditions under which these effects emerge remain to be tested. The inconsistent findings regarding arousal’s impact on time perception as indexed by pupil size also call for further empirical investigation. By testing these influences separately, we aim to contribute to a more nuanced understanding of time perception, challenging and potentially refining existing theoretical models. To that end, we employed a temporal bisection task in two complementary experiments, varying both the brightness of the stimulus (Experiment 1) and the background against which it was timed (Experiment 2). Our results showed that the effect of brightness on time perception is such that increased brightness results in longer perceived durations, and that, crucially, this is the case for both the brightness of the to-be-timed stimulus and the background on which this stimulus is presented. Additionally, in both experiments we tracked spontaneous fluctuations of the pupil size as a measure of arousal. We expected larger pupils to be associated with longer perceived durations. However, against our expectation, our results suggest that spontaneous pupil size is unrelated to perceived time.

## Experiment 1

### Methods

#### Participants

Thirty participants (25 women; *M*_age_ = 22.9 years) were included in Experiment 1. Participants received a compensation of €8 for their contribution. Participants provided written informed consent prior to the study. All participants had normal or corrected-to-normal vision. No participants were excluded from the analyses. In the absence of an expected effect size, sample size was based on previous similar studies on temporal perception.

#### Stimuli and apparatus

**Fig. 1:**
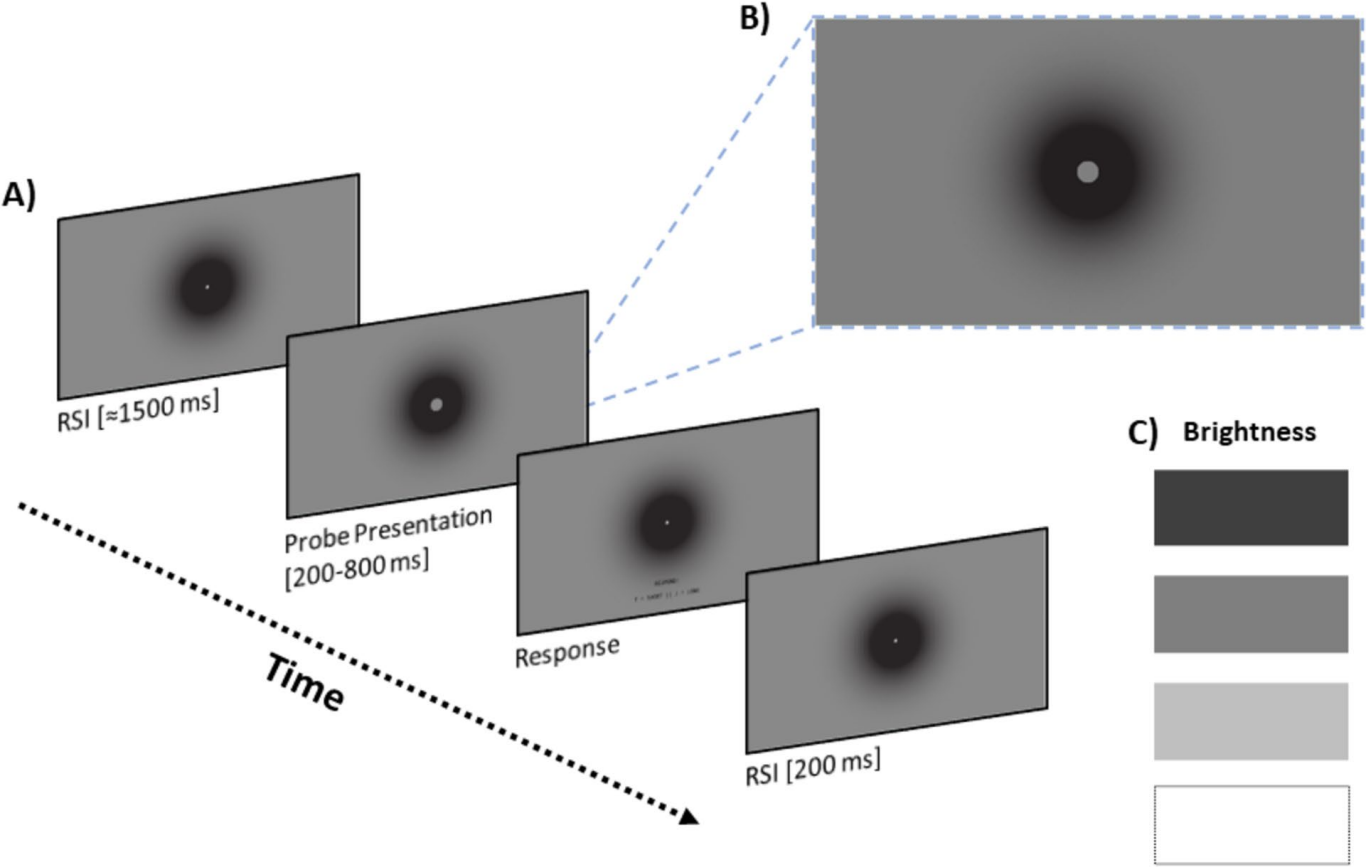
**A** Timeline of a single trial in experiment 1. **B** Sample screen depicting the background gradient in a trial with the annulus surrounding the fixation dot during stimulus presentation. **C** Four levels of stimulus brightness (top to bottom; low, medium, high, maximum/white)

Stimuli were presented on a 27-in. LCD monitor (1,920 × 1,080 pixels; 60 Hz) using OpenSesame 3.3 (Mathôt & March, 2022; Mathôt et al., 2012) with the PsychoPy (Peirce, 2007) framework for stimulus presentation and PyGaze (Dalmaijer et al., 2014) for eye tracking. An EyeLink 1000 (SR Research) recorded the right eye at 1000 Hz. The flat-screen monitor was placed approximately 60 cm from a chin rest stabilized on the edge of the supporting desk. Participants provided their responses using a wired keyboard while their heads were stabilized on the chin rest for the duration of the experimental session. The to-be-timed stimuli consisted of a filled circle with various levels of brightness (discussed below) briefly presented in the center of the screen (subtending a visual angle of 2.39°), which occluded a white central fixation dot (0.48°). The central area of stimulus presentation was fully black (9.90°), transitioning as a gradient to a gray circular background towards the edge of the screen (≈24°) in order to minimize contrast effects (Karsilar et a., 2024; see Fig. 1B).

#### Procedure

Each session consisted of a variable-brightness block, followed by a shorter constant-brightness block. The presentation duration and the brightness of the timed stimuli were randomly varied in the variable-brightness block, whereas stimulus brightness was fixed in the constant-brightness block. An entire session with the two blocks lasted approximately 50 min. Both blocks started with a 9-point eye-tracking calibration with white targets. Each trial in the session was initiated with a drift correction (single-point recalibration) procedure. Participants were told that the purpose of the eye tracker was to ensure that they always focused on the central fixation dot throughout the experiment.

#### Temporal bisection procedure

##### Training

The temporal bisection task is a form of a two-alternative forced-choice task that entails categorizing experienced durations as “short” or “long” based on their subjective similarity to previously learned short and long reference durations (Penney & Cheng, 2018; Wearden & Ferrara, 1995). As such, the task started with the presentation of the two reference durations (200.4 and 801.6 ms) in the form of the aforementioned circle with a constant brightness of 15.8 cd/m^2^, followed by a training session. During training, the two reference durations were presented randomly with equal likelihood. Participants’ pressed the “F” and “J” keys with their index fingers to report a short and a long duration, respectively. Following common recommendations in the literature, participants were instructed not to count or use any other chronometric methods, such as tapping, throughout the experiment. Participants were instructed to only attend to the amount of time that the circle stayed on the screen and decide which of the previously presented durations it matched. The training phase was terminated upon 20 correct responses in the variable-brightness block, and 10 correct responses in the constant-brightness block. Given the high discriminability between the two reference durations, no feedback was given during training. The majority of the participants required fewer than four correction trials.

##### Test

The test phase was identical to the training phase, except for the presentation of four durations (317.3, 434.2, 567.8, 684.7 ms) in addition to the two reference durations (200.4 and 801.6 ms). Participants were explicitly provided with this information and were asked to decide which of the two reference durations they learned during the training phase was closest to the one being presented on each trial. A typical trial consisted of a response-to-stimulus (RSI) interval between 1,000 ms to 2,000 ms, followed by the probe presentation and a response screen with no explicit deadline. Each response was followed by a fixed 200 ms interval (Fig. 1A). The RSI was randomized in or to preclude the participants from establishing a rhythmic response pattern in the session. No feedback was given. All participants were observed to readily follow the instructions of the test phase. During the test phase of the variable-brightness block, the brightness of the timed stimulus was also manipulated trial-to-trial (four levels; low, medium, high, maximum/white; 5.5, 15.8, 35.6, and 66 cd/m^2^, respectively; see Fig. 1C). We selected the four brightness values such that they would be logarithmically spaced between 5.5 and 66 cd/m^2^ to align with the Weber–Fechner law, which models perception as proportional to the logarithm of stimulus intensity. This approach ensures perceptually meaningful spacing while maintaining a straightforward model for grayscale representation. In order to achieve a comprehensive mapping of duration discriminability and to control for the effect of ensemble statistics and potential carryover effects, we employed a De Bruijn sequence in designing the temporal bisection task. This generated sequence ensured that each of the six probe durations were preceded and followed by every other duration in the set an equal number of times. The length of the shortest De Bruijn sequence of a set of *n* elements is *n*^2^. Thus, in order to ensure that each of the four stimulus brightness levels were presented an equal number of times, a single De Bruijn sequence of six probe durations required 144 trials. In order to achieve a better fit to data, each probe duration was presented 12 times by doubling the amount of trials, resulting in a test block of 288 trials in total, which also fit well with the targeted session duration of 50–60 min.

#### Data processing and analysis

Following preprocessing in Python, data were analyzed using generalized linear mixed-effects models. Analyses were conducted using the “glmer” function in the *lme4* package (Bates et al., 2015) in R (R Core Team, 2020). To facilitate interpretation and improve numerical stability, the probe duration variable was mean-centered (but not *z*-scored) before all analyses. Bayesian and frequentist statistical analyses were performed using JASP software (JASP Team, 2023). To control for response artifacts, trials with reaction times shorter than 100 ms or longer than 5,000 ms were excluded.

##### Pupil data processing

Preprocessing of EyeLink 1000 data was performed using the *eyelinkparser* package (Mathôt & Vilotijević, 2023) in Python. Pupil size, originally recorded in arbitrary units, was converted to mm based on a conversion formula previously established for our setup. Missing or invalid data were interpolated if possible and removed otherwise. Invalid trials were defined based on standard pupillometry preprocessing as follows: (1) trials with extreme baseline pupil sizes (*z*-score > 2 or < − 2) were excluded​; (2) trials where more than 50% of the pupil data were missing due to blinks, tracking loss, or artifacts were removed; and (3) trials with extreme pupil-size deviations (more than ± 3 *SD* from the mean) or unnatural jumps were also excluded (see Mathôt & Vilotijević, 2023). 

##### Choice of analytical approach

 Traditional psychophysical approaches to time perception often rely on fitting psychometric functions (e.g., sigmoid or Weibull curves) to response proportions and extracting the point of subjective equality (PSE; Bausenhart et al., 2018). While widely used, these methods are limited by their reliance on predefined functional forms and parametric response distributions, which can introduce biases in PSE estimation (Grondin, 2024). Additionally, aggregating data across trials leads to a loss of trial-level variability and inadequately accounts for both intra- and interindividual differences, making it difficult to handle hierarchical data structures effectively (García-Pérez, 2014a, b; Vidotto et al., 2019; Wang & Reynaud, 2023). Consequently, generalized linear mixed models (GLMM) have been increasingly recommended in time perception research for their ability to improve estimation accuracy and generalizability (García-Pérez, 2014a, b; Stone, 2014). GLMMs offer multiple advantages (V. A. Brown, 2021): They retain all trial-level data, enhancing statistical power and sensitivity; provide a suitable framework for binomial dependent variables, addressing the nonnormality inherent in timing tasks distinguishing “long” versus “short” responses (Wang & Reynaud, 2023); and are robust to missing and unbalanced data (e.g., excluded trials due to badly recorded pupils), reducing biases from trial exclusions common in psychophysical studies (Vidotto et al., 2019). Moreover, GLMMs allow for the direct estimation of experimental effects on temporal decision-making without requiring an intermediate curve-fitting step (García-Pérez, 2014a, b; Wang & Reynaud, 2023). Their hierarchical structure enables the modeling of both within- and between-subject variability, offering a comprehensive analysis of perceptual decision-making. Recent methodological work further supports the use of GLMM in psychophysical research, particularly when trial-level response variability carries meaningful information, such as that observed in the temporal bisection task (García-Pérez, 2014a, b; Wang & Reynaud, 2023).

To address these issues, we employed a GLMM approach to analyzing our data, enabling trial-level analysis while incorporating subject-specific random effects. We fit a logistic regression model predicting the probability of a “long” response as a function of probe duration and other experimental predictors. The model, using a binomial family with a “logit” link function, was optimized via maximum likelihood estimation with the Laplace approximation and the *bobyqa* optimizer. By including both random intercepts and slopes, GLMMs capture interindividual variability and model the influence of experimental factors, such as brightness or pupil size, on time perception more effectively than traditional methods (Grondin, 2008).

In practice, we compared two variants of this model: a “simple” model with only a random intercept per participant, and a more “complex” one that added random slopes for both probe duration and each predictor in order to provide a more comprehensive picture of the data. The simpler “random-intercepts-only” model accounts for individual differences in overall response bias (i.e., the baseline tendency to respond “long”) but assumes that the effect of probe duration and the predictor (e.g., brightness) is the same for every participant. The more complex model additionally tests whether participants differ in how strongly these factors influence their responses. While such complexity can more accurately capture true interindividual variability, it also carries the risk of overparameterization or convergence problems if the dataset is not sufficiently large or if effects do not meaningfully vary across individuals. Therefore, we include both models in our analyses, and we explicitly report any convergence warnings or boundary fits as they arise.

Lastly, to validate the robustness of our GLMM results, we extracted summary parameters for each model, which were then analyzed by an analysis of variance (ANOVA; both Bayesian and frequentist). The first parameter of interest, PSE, represents the temporal discrimination threshold and is determined statistically as the duration at which a participant is equally likely to give both a “short” and a “long” response. PSEs were computed by fitting psychometric curves to each participant’s data using parameters obtained from the GLMM and identifying the probe duration at which the predicted probability of a “long” response equaled 0.5 for each participant. A lower PSE for a given condition therefore represents a longer perceived duration compared with a higher one, and vice versa. Second, in order to detect any potential difference in stimulus discriminability as a function of a predictor (i.e., brightness or pupil size), we also calculated Weber Ratios (WR). The WR, computed as the ratio of the difference limen (half the distance between the first and the third quartiles: ([p(long) = 0.75 – p(long) = 0.25]/2) to the PSE, provides a measure of perceptual sensitivity to temporal distortions across conditions. A higher WR implies lower temporal sensitivity and precision, *ceteris paribus*. However, given the methodological advantages of GLMM described above, we consider it the more appropriate analytical approach for elucidating the effect of brightness and pupil size on time perception as employed in this study.

### Results

#### Bright stimuli are perceived as longer

**Fig. 2:**
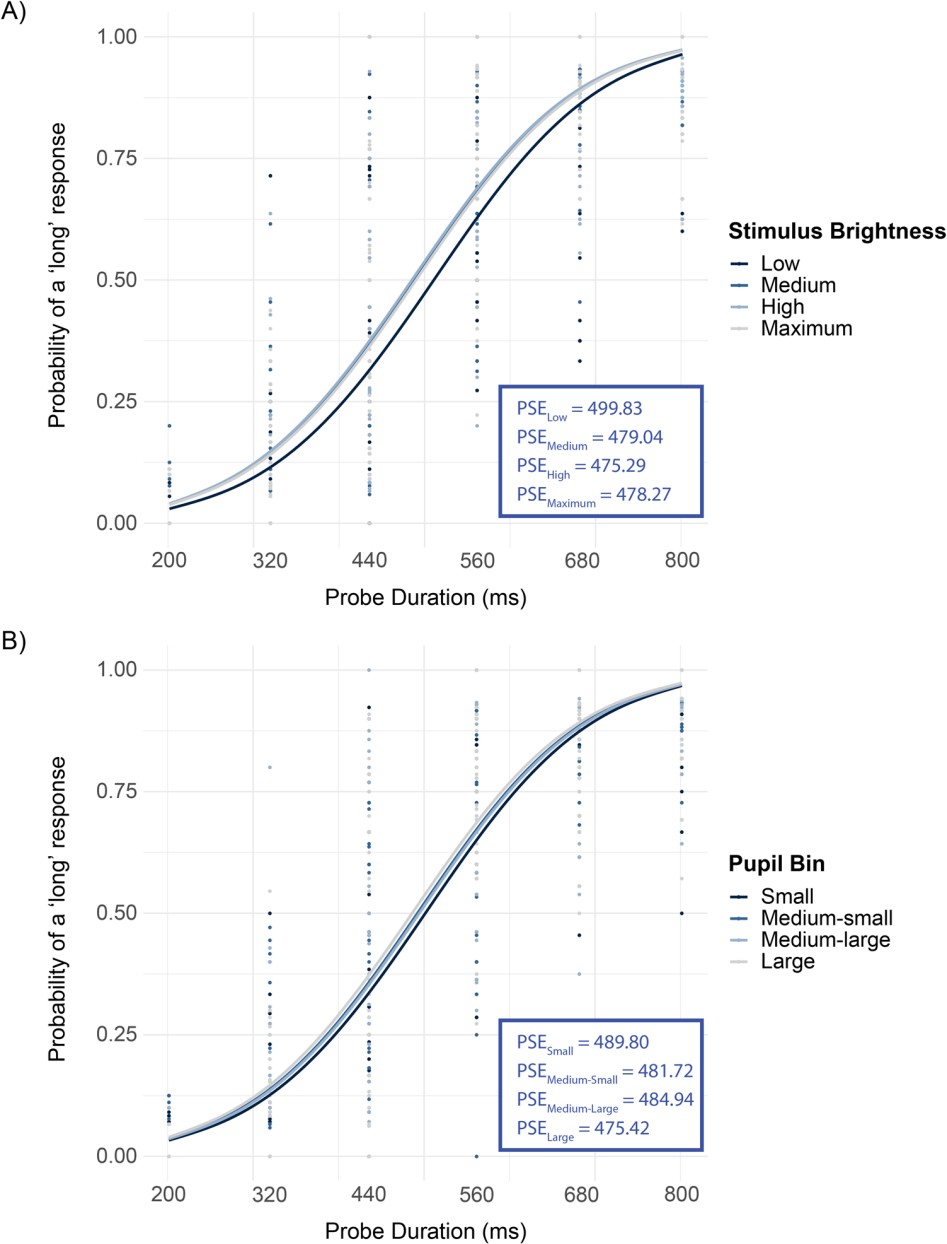
Average responses for individual observers (dots) and model-predicted probabilities (solid lines) from a generalized linear mixed-effects model with a logistic link function, plotted as a function of probe duration for each level of (**A**) stimulus brightness and (**B**) pupil bin in Experiment 1. PSE values are given as insets

To assess the influence of stimulus brightness on perceived durations, we evaluated a GLMM with fixed effects for probe duration and stimulus brightness, along with a random intercept for individual subjects. The low stimulus brightness condition was set as the reference category. Fixed effects in this intercept-only, or “simple,” model demonstrated a highly significant effect of probe duration (β = 12.604, *SE* = 0.261, *p* < 0.001; Fig. 2). With regard to the effect of stimulus brightness, medium brightness (β = 0.277, *SE* = 0.095), high brightness (β = 0.323, *SE* = 0.095), and maximum (white) brightness (β = 0.262, *SE* = 0.096) were all significantly associated with higher log-odds of a long response compared with the low brightness (all *p* < 0.01). The effect for the intercept was nonsignificant (*p* > 0.05). To verify the robustness of our findings from the GLMM, we conducted additional repeated ANOVAs on PSE values obtained via the method described above. The results qualitatively replicated our GLMM findings, confirming a highly significant effect of brightness on PSEs, *F*(1.98, 59.25) = 146,334.34, *p* < 0.001, ω^2^ₚ = 0.014, BF_10_ = 1.0 (see Fig. 2A).

As mentioned above, we also tested a more complex model which included random slopes for all fixed effects. The results were similar to the simpler model; fixed effects in the complex model also showed a significant effect for the probe duration (β = 13.417, *SE* = 0.615, *p* < 0.001). Medium brightness (β = 0.288, *SE* = 0.122), high brightness (β = 0.337, *SE* = 0.147), and maximum brightness (β = 0.265, *SE* = 0.137) also remained significant predictors (all *p* values < 0.05). The effect for the intercept was nonsignificant (*p* > 0.05). Both models were validated by obtaining an identical pattern of results through split-half testing (results not shown). Although the more complex model provided a more accurate fit to data (Akaike information criterion [AIC] = 5797.9, Bayesian information criterion [BIC] = 5924.7) compared with the simpler model (AIC = 5867.9, BIC = 5910.1), the significance and directionality of the fixed effects remained consistent between both models.

Taken together, the results show that bright stimuli are perceived as longer, where qualitatively the biggest difference exists between the lowest level of brightness and all other levels of brightness.

#### No clear correlation between arousal-related pupil-size changes and perceived duration

To assess the relationship between pupil size and perceived duration, a similar pair of simple and complex GLMMs were tested, with pupil bin as the fixed effect, replacing stimulus brightness in the models detailed above. Here, “pupil bin” was determined as described below to reflect endogenous, or spontaneous, fluctuations in pupil size, which are assumed to reflect fluctuations in arousal. In order to be readily comparable to the model explaining the effect of four stimulus brightness levels above, average pupil sizes during the 900–1,000 ms window of the RSI interval on each trial were binned into four roughly equal-sized categories (small, medium-small, medium-large, large).

##### Variable-brightness block

The binning operation in the variable-brightness block was performed separately for each level of stimulus brightness in order to avoid conflating the effect of brightness on baseline pupil size with spontaneous fluctuations in pupil size. Crucially, since higher-luminance stimuli can induce a direct (and almost immediate) pupil constriction and thus potentially shift the overall level of pupil size, we wanted to cleanly separate “spontaneous” or endogenous fluctuations in pupil size from any brightness-driven pupil responses. To do so, for each level of stimulus brightness (e.g., low, medium, high, maximum), we created an internal distribution of baseline pupil sizes across trials in that brightness level alone and then binned those baseline pupil sizes into four equally sized categories (small, medium-small, medium-large, large). This approach ensures that a “large” pupil within a low-luminance context is categorized relative to other trials in the low-luminance condition, rather than relative to a high-luminance condition. By separately binning within each brightness level, we avoided conflating any systematic brightness effect on pupil diameter with the spontaneous, trial-to-trial variation in arousal. Once the four bins were assigned within each brightness condition, we pooled all trials—now each labeled with a pupil bin—into one dataset for the main logistic regression. This way, the pupil bin factor reflected true endogenous variability rather than luminance-induced variability. Pupil bins from the four brightness levels were then entered into the models. As with the models with stimulus brightness described above, a simple and a complex model were fitted to data, with the latter allowing for random slopes for the effect of pupil bin and probe duration. The small pupil bin condition was set as the reference category. In the simple model (AIC = 5877.8, BIC = 5920.0), the fixed effects revealed a nonsignificant effect for the intercept (*p* > 0.05), whereas probe duration demonstrated a significant effect on choice likelihood (β = 12.587, *SE* = 0.260, *p* < 0.001). Regarding the effect of pupil size, the Large pupil bin was found to have a significant effect when compared to the reference category (β = 0.195, *SE* = 0.096, *p* = 0.0418). However, the other two pupil bins (medium-small and medium-large) did not have a significantly different effect than the reference category (both *p* values > 0.05). The complex model (AIC = 5877.8, BIC = 5920.0) also yielded a nonsignificant result for the intercept (*p* > 0.05) and a significant effect for probe duration (β = 13.310, *SE* = 0.607, *p* < 0.001). Concerning the effect of pupil size, identical with the simple model, the large pupil bin showed a marginally significant effect compared to the reference category (β = 0.20102, *SE* = 0.10460, *p* = 0.0546), while the effects of the other two larger pupil bins were not significant (both *p* values > 0.05). Notably, the complex model exhibited a boundary fit, which suggests that the model was overparameterized, implying that the additional complexity in this model may not have been warranted by the pupil size data.

##### Constant-brightness block

As with the variable-brightness block, pupil sizes were categorized into four approximately equal-sized bins (small, medium-small, medium-large, large) for the constant-brightness block. The two model structures (simple and complex) with the small pupil bin condition as the reference were consistent with those reported previously. For the simple model (AIC = 1457.1, BIC = 1491.0), fixed effects revealed that, both the intercept (β = 0.571, *SE* = 0.238, p = 0.0164), and the probe duration were significant (β = 13.239, *SE* = 0.562, *p* < 0.001). With regard to the effect of pupil size, compared to the Small pupil bin (reference category), none of the larger three bins were associated with higher probability of a long response (all *p* values > 0.05). For the complex model (AIC = 1440.3, BIC = 1542.0), fixed effects were largely consistent with the simple model. The intercept (β = 0.867, *SE* = 0.274, *p* = 0.002), and the probe duration remained significant (β = 14.907, SE = 1.104, p < 0.001). As with the variable-brightness block, the complex model in the constant-brightness block also exhibited a boundary (singular) fit.

Taken together, except for a marginally significant difference in one of several contrasts (large vs. small pupils in the variable-brightness block), the results do not show a convincing relationship between pupil size and perceived duration.

#### No effects of stimulus brightness and pupil size on duration discriminability

Lastly, we tested the effect of stimulus brightness and pupil size on duration discriminability. One-way ANOVAs showed no effect of stimulus brightness (*p* = 0.826; BF_10_ = 0.063) on WR. Similarly, there was no effect of pupil bin in the variable-brightness block (*p* = 0.221; BF_10_ = 0.241) or the constant-brightness block (*p* = 0.634; BF_10_ = 0.086).

Overall, results suggest no effect of stimulus brightness or pupil bin on stimulus discriminability in Experiment 1.

## Experiment 2

Experiment 1 demonstrated a robust effect of stimulus brightness on perceived durations, with no clear relationship between pupil size and the likelihood of a “long” response, suggesting that arousal did not, in this context, affect perceived duration. Experiment 2 aimed to test whether this effect is specific to the brightness of the to-be-timed stimulus, or whether it generalizes to the brightness on which the stimulus is presented. As such, the brightness manipulation of the timed stimuli in Experiment 1 was replaced by a brightness manipulation of the background on which the stimuli were presented. All data preprocessing stages, and modeling procedures were identical to Experiment 1.

### Methods

#### Participants

Thirty participants (23 women; *M*_age_ = 20.95 years) were included in Experiment 2. Participants received course credit for their contribution. All participants provided written informed consent prior to the study. All had normal or corrected-to-normal vision. No participants were excluded from the analyses. Sample size was matched to Experiment 1.

#### Stimuli and apparatus

**Fig. 3:**
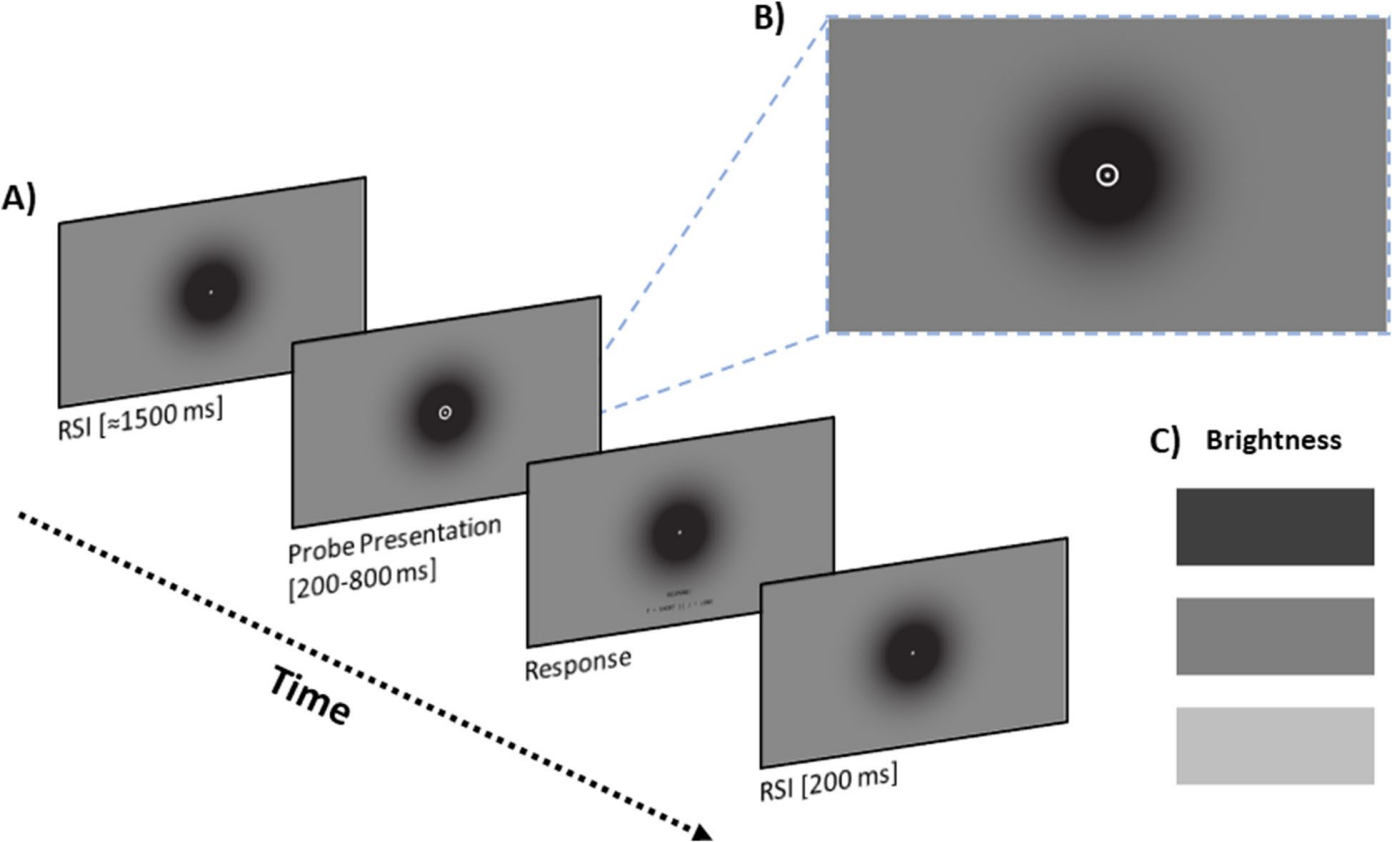
**A** Timeline of a single trial in Experiment 2. **B** Sample screen depicting the background gradient in a trial with the annulus surrounding the fixation dot during stimulus presentation. **C** Three levels of background brightness (top to bottom; low, medium, high brightness)

The apparatus was identical to Experiment 1. The to-be-timed stimulus in Experiment 2 consisted of an annulus briefly presented in the center of the screen (*R*_inner_ = 1.62°, *R*_outer_ = 2.39°), which surrounded a white central fixation dot (Fig. 3B). As with Experiment 1, the central area of the stimulus presentation was fully black, transitioning as a gradient to a gray background towards the edge of the screen.

#### Procedure

The procedure was identical to Experiment 1, except that we now manipulated the brightness of the background, rather than that of the probe (Fig. 3C). Crucially, the color of the timed stimulus was white, and was constant throughout the session (Fig. 3A). A session with the two blocks lasted approximately 45 min. All remaining details pertaining to the eye tracking procedure were identical to Experiment 1.

#### Temporal bisection procedure

The parameters and the task demands of training and test phases of the temporal bisection procedure were identical to those in Experiment 1. The annulus detailed above was used as the training and the test stimulus. As with Experiment 1, the majority of the participants required less than four correction trials during training. The brightness of the background during the test phase was manipulated trial-to-trial by varying values in the red–green–blue color space (three levels; low, medium, high; 5.5, 15.8, 35.6 cd/m^2^). A white background was not added due to the expected intensity of the effect. As with Experiment 1, we employed a De Bruijn sequence in designing the temporal bisection task, resulting in a test block of 216 trials in total.

### Results

#### Stimuli on a bright background are perceived as longer

**Fig. 4:**
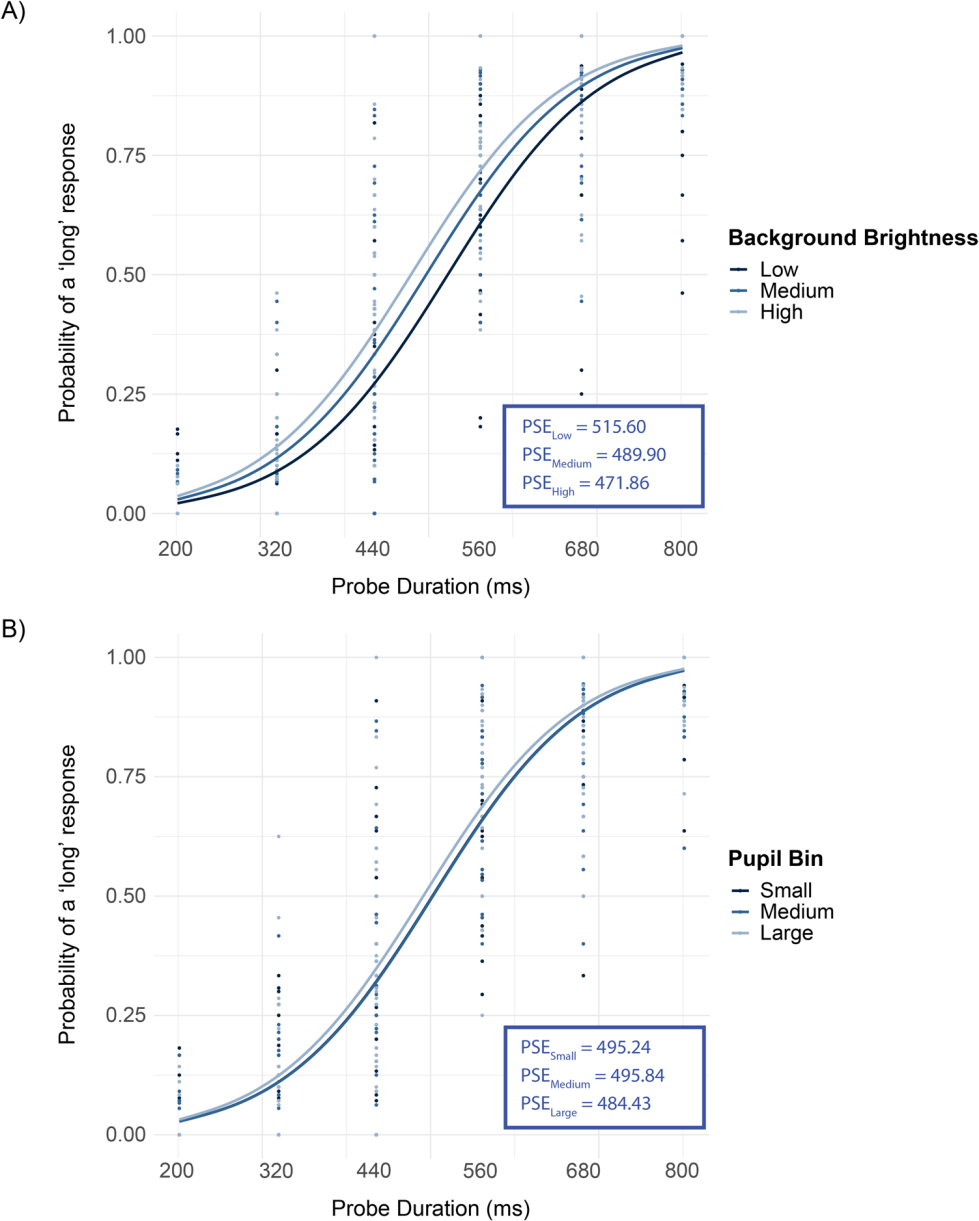
Average observed responses for individual observers (dots) and model-predicted probabilities (solid lines) from a generalized linear mixed-effects model with a logistic link function, plotted as a function of probe duration for each level of (**A**) background brightness and (**B**) pupil bin in Experiment 2. PSE values are given as insets

The analysis procedure was identical to that of Experiment 1. The fixed effects in the simple model displayed a non-significant trend for the intercept (*p* > 0.05), whereas the probe duration showed a significant effect (β = 12.737, *SE* = 0.302, *p* < 0.001; Fig. 4). Compared with the low brightness condition, both medium brightness (β = 0.327, *SE* = 0.096) and high brightness (β = 0.557, *SE* = 0.096) increased the likelihood of a prolonged response (both *p* values < 0.001). In the complex model, similarly the intercept was nonsignificant (*p* > 0.05), but the probe duration’s influence was evident (β = 13.627, *SE* = 0.676, *p* < 0.001). The associations of medium (β = 0.318, *SE* = 0.130) and high brightness (β = 0.560, *SE* = 0.111) remained robust when juxtaposed with the low brightness baseline (both *p* values < 0.05). Comparative analysis based on AIC and BIC values showed the complex model to be a more precise representation of the data (AIC = 4334.1, BIC = 4421.9) than the simple model (AIC = 4391.9, BIC = 4425.7), although the significance and general direction of the fixed effects were consistent across both modeling approaches. To verify the robustness of our findings from the GLMM, we conducted additional repeated ANOVAs on PSE values. As with Experiment 1, the results qualitatively replicated our GLMM findings, confirming a highly significant effect of brightness on PSEs, *F*(2, 58) = 704,046.34, *p* < 0.001, ω^2^ₚ = 0.077, BF_10_ = 1.0 (see Fig. 4A).

Taken together, the results show that stimuli are perceived as longer when they are presented on a bright background.

#### No clear correlation between arousal-related pupil-size changes and perceived duration

To test the relationship between spontaneous pupil size and perceived duration, we conducted the same analysis as we did for Experiment 1, except that we created three pupil bins (instead of four) to match the number of different brightness levels in Experiment 2.

##### Variable-brightness block

For the simple model (AIC = 4423.4, BIC = 4457.2), the fixed effects highlighted a nonsignificant effect for the intercept (*p* > 0.05), whereas the probe duration was a significant predictor (β = 12.658, *SE* = 0.299, *p* < 0.001). In relation to pupil size effects, as contrasted with the Small pupil bin reference, neither of the medium or large pupil bins manifested significant capability in predicting the likelihood of a long response (both *p* values > 0.05). With the complex model (AIC = 4370.7, BIC = 4458.5) the results provided a similar narrative. The intercept in the complex model was not statistically significant (*p* > 0.05), while the probe duration retained its significant predictive power (β = 13.489, *SE* = 0.674, *p* < 0.001). Mirroring the findings from the simple model, pupil size did not offer any significant predictive power when set against the reference category (both *p* values > 0.05). Notably, the complex model indicated a boundary (singular) fit, suggestive of an overparameterized model, underscoring the possibility that the complexity in this model might not be adequately supported by the pupil size data.

##### Constant-brightness block

The fixed effects of the simple model revealed the intercept to be marginally significant (β = 0.3532, *SE* = 0.2472, *p* = 0.153). The probe duration, as in Experiment 1, remained significant (β = 13.2684, *SE* = 0.5557, *p* < 0.001). Concerning the effect of pupil size, compared to the reference category (small bin), the likelihood of a long response was not significantly different for the medium or the large pupil bin (both *p* values < 0.05). The complex model failed to converge.

Taken together, the results do not show any relationship between pupil size and perceived duration.

#### No effects of background brightness and pupil size on duration discriminability

Like Experiment 1, we compared WR values (ratio of DL to PSE) for different brightness levels and pupil bins in Experiment 2. One-way ANOVAs showed no effect of background brightness (*p* = 0.424; BF_10_ = 0.198) or an effect of pupil bin in the variable-brightness block (*p* = 0.33; BF_10_ = 0.242) or the constant-brightness block (*p* = 0.191; BF_10_ = 0.385) on WR. Overall, identical to those in Experiment 1, results in Experiment 2 suggest no effect of background brightness or pupil bin on stimulus discriminability.

### Discussion

Although people use duration information effectively to guide behavior, subjective time perception often diverges from veridical time (Eagleman, 2008; Zakay & Block, 1997). Research shows that both cognitive states and physical event characteristics impact duration perception (de Hevia et al., 2014; Kanai et al., 2006; Kaneko & Murakami, 2009; Lake et al., 2016; Vidaud-Laperrière et al., 2022; Walsh, 2003). In this study, we show that increased brightness results in longer perceived durations, and surprisingly, that this happens regardless of whether the brightness of a to-be-timed stimulus itself or its background is manipulated. We further show that arousal as indexed by pupil size does not notably affect perceived duration.

We reported two experiments aimed to clarify the specifics of the mechanism through which brightness influences perceived time. The first experiment demonstrated a direct effect of stimulus brightness on perceived durations, while the second experiment, which kept stimulus brightness constant and varied background brightness, showed similar results. These findings challenge the assumption that brightness affects time perception solely through the stimulus properties, instead suggesting a broader influence of environmental luminance. Specifically, the results suggest that the pulse rate of a hypothetical internal clock may be affected by brightness very generally and irrespective of the source of the brightness. In other words, the results suggest that the internal clock may be driven, among other things, by low-level visual input.

Within the context of an information-processing model, temporal integration is thought to be accelerated in response to increased stimulus intensity, which would be captured by an increased tendency to report a duration as “long” (Allan & Gibbon, 1991; Wearden, 1991). This tendency was observed in both of our experiments. These results can be explained by an accelerated internal clock mediated by increased stimulus intensity (i.e., higher brightness; Ivry & Schlerf, 2008; Penney et al., 2000; Treisman, 1963). This shift, evident in our experiments, suggests a stimulus-driven acceleration of the internal timing mechanism. In some ways the effect of stimulus intensity is analogous to the effect of arousal (if any, see below), in the sense that stimulus intensity affects the intensity of low-level visual processing, whereas arousal reflects the intensity of higher-level cognitive processes. However, it is important to note that shifts in PSEs are likely a culmination of several cognitive processes working in concert—including that of temporal attention—therefore attributing these results solely to increased stimulus intensity would likely fall short of capturing the underlying complexity of the observed effect (Angrilli et al., 1997; Droit-Volet & Meck, 2007; Gil & Droit-Volet, 2012; Mella et al., 2011; Smith et al., 2011; Wearden et al., 1998). Furthermore, recent findings suggest that the extent to which stimulus features influence time perception depends on the task used to measure the effect. Specifically, Petrizzo et al. (2023) demonstrated that numerosity biases duration judgments only in discrimination tasks (e.g., two-alternative forced choice), but not in equality judgments or reproduction tasks. Given that our study employed a temporal bisection task, which similarly requires categorical judgments, it is possible that decision-related processes contributed to the observed effect of brightness on time perception. Future research could explore whether these effects persist in noncategorical timing tasks (e.g., duration reproduction or equality judgments) to disentangle purely perceptual influences from those arising from top-down decisional factors such as heuristic-based decision-making strategies and biases.

It is not straightforward to explain our results through an effect of attention mediated by changes in brightness (Block & Gruber, 2014; Coull et al., 2004; Zakay & Block, 1997). Research indicates that attending to nontemporal task demands competes with focusing temporal elements (Brown, 1997; Zakay & Block, 1997). Since all attention lapses, whether intentional or not, can decrease the probability of registering time-representative pulses, this competition can lead to shorter perceived durations (Casini & Macar, 1997; Fortin & Masse, 2000; Grondin, 2010; Zakay & Block, 1997). Possibly, in Experiment 1, participants attended more to the stimulus if it was brighter, which could indirectly lead to increased perceived duration of bright stimuli; in this interpretation, the effect of brightness of perceived duration would be mediated by temporal attention. However, in Experiment 2, participants likely attended *less* to the stimulus if it was presented on a brighter background, which in this interpretation should lead to reduced perceived duration—the opposite of what we found. Thus, our findings likely reflect effects of brightness rather than attentional changes.

In both experiments we also monitored spontaneous pupil size to explore its potential impact on time perception for a given level of stimulus or background brightness. The motivation for this inquiry was threefold. Firstly, we aimed to utilize the established link between arousal and pupillary response, given arousal’s presumed effect on extending perceived time durations. Secondly, viewed through the lens of the “coding efficiency model” of temporal perception—a theory proposing that our brains optimize the processing of temporal information to enhance efficiency (Eagleman & Pariyadath, 2009)—higher neural activation on the retina would lead to spontaneously larger pupils being associated with longer perceived durations—due to higher light influx (Matthews & Meck, 2016). Lastly, research on the effect of neuromodulators such as dopamine and serotonin have postulated a common pathway for pupillary dynamics and temporal information processing, further bolstering a potential correlation between the two (Aston-Jones & Cohen, 2005; Berridge & Waterhouse, 2003; Buhusi & Meck, 2005; Coull et al., 2011; Joshi et al., 2016; Meck, 2006). Surprisingly, neither experiment found a significant correlation between pupil size and time perception, a finding that diverges from existing, albeit limited, literature. This suggests that, at least in the present paradigm, perceived duration is not affected by arousal.

Nevertheless, there are several methodological considerations that may have influenced our findings regarding the null effect of pupil size on duration perception. First, we measured naturally occurring (as opposed to experimentally induced) pupil-size variations, which might generate only modest changes in arousal insufficient to impact timing performance (Joshi & Gold, 2020). Second, the pronounced effect of brightness may have overshadowed subtle pupil-driven effects, particularly given that pupil diameter is itself partially governed by luminance levels (Binda et al., 2013; Mathôt & Van der Stigchel, 2015). Third, despite our trial-level analyses and relatively large number of trials per participant, we cannot rule out that a more targeted manipulation of arousal (e.g., emotional stimuli, reward) could reveal a clearer pupil–time link (Akdoğan et al., 2016; Einhauser et al., 2010; Goldinger & Papesh, 2012; Naber et al., 2013; Rohenkohl et al., 2012; Suzuki et al., 2016; Van Rijn et al., 2012). Additionally, while our generalized linear mixed models were robust and accounted for various sources of variability, it is possible that alternative analytical approaches or more sensitive measures of arousal might detect effects that our current analyses did not. Nonetheless, our paradigms successfully captured large, systematic shifts in timing judgments due to brightness, suggesting that our design was sufficiently sensitive to detect moderate effect sizes. Therefore, while we cannot definitively rule out a role for arousal in time perception, our data suggest that any effect of spontaneous pupil-size fluctuations on duration judgments, at least under the present conditions, is minimal. Lastly, one limitation of our study is that we assumed brightness perception follows the Weber–Fechner law rather than measuring subjective brightness levels. Future studies could improve upon this by having participants rate brightness levels beforehand and then using those ratings to calibrate stimuli (see Hurvich & Jameson, 1966; Palmer, 1999; Sulutvedt et al., 2021 for related work on brightness perception). This would better account for individual differences and enhance sensitivity to potential pupil-linked effects on time perception.

In conclusion, the combined behavioral and pupillometric findings presented here help illuminate the intricate interplay between sensory input and temporal cognition (Laeng et al., 2012; Matthews & Meck, 2016). More specifically, the findings that increased brightness, be it of the stimulus or its background, lengthens perceived duration, while spontaneous pupil size shows no significant correlation with time perception, challenge models that underpin widely held assumptions regarding a directional relationship between “arousal” and time perception. Our research thus puts forward a more nuanced view where low-level visual properties significantly influence our perception of time, independent of arousal-linked mechanisms traditionally associated with pupil size. Specifically, the finding that the brightness of the background affects time perception in more or less the same way as the brightness of the timed stimulus underscores the idea that our cognitive evaluation of time is holistic and integrative, relying not merely on the properties of the timed stimuli but on a composite of sensory experiences (Calvert et al., 2004; Driver & Noesselt, 2008; Matthews, 2011; Stein & Meredith, 1993; Stein et al., 2009; van Wassenhove, 2009; van Wassenhove et al., 2008). As time perception remains a central dimension of our conscious experience, unraveling its multifaceted nature will be instrumental in painting a more complete picture of human cognition.

## Data Availability

Experimental data, the experiment script, and analysis scripts can be found online (https://osf.io/9gkbw/).

## References

[CR1] Akdoğan, B., Balcı, F., & van Rijn, H. (2016). Temporal expectation indexed by pupillary response. *Timing & Time Perception,**4*(4), 354–370.

[CR2] Allan, L. G. (1979). The perception of time. *Perception & Psychophysics,**26*(5), 340–354.

[CR3] Allan, L. G., & Gibbon, J. (1991). Human bisection at the geometric mean. *Learning and Motivation,**22*(1), 39–58.

[CR4] Allman, M. J., Teki, S., Griffiths, T. D., & Meck, W. H. (2014). Properties of the internal clock: First- and second-order principles of subjective time. *Annual Review of Psychology,**65*, 743–771. 10.1146/annurev-psych-010213-115117 24050187

[CR5] Angrilli, A., Cherubini, P., Pavese, A., & Manfredini, S. (1997). The influence of affective factors on time perception. *Perception & Psychophysics,**59*, 972–982. 9270369 10.3758/bf03205512

[CR6] Aston-Jones, G., & Cohen, J. D. (2005). An integrative theory of locus coeruleus-norepinephrine function: Adaptive gain and optimal performance. *Annual Review of Neuroscience,**28*, 403–450. 10.1146/annurev.neuro.28.061604.135709 16022602

[CR7] Bates, D., Maechler, M., Bolker, B., Walker, S., Christensen, R. H. B., Singmann, H., ... & Bolker, M. B. (2015). Package ‘lme4’. *Convergence, 12*(1), 2.

[CR8] Basgol, H., Ayhan, I., & Ugur, E. (2021). Time perception: A review on psychological, computational, and robotic models. *IEEE Transactions on Cognitive and Developmental Systems,**14*(2), 301–315.

[CR9] Bausenhart, K. M., Di Luca, M., & Ulrich, R. (2018). Assessing duration discrimination: Psychophysical methods and psychometric function analysis. In F. Balci, Á. Correa, M. Di Luca, & A. Vatakis (Eds.), *Timing and time perception: Procedures, measures, & applications* (pp. 52–78). Brill.

[CR10] Beatty, J., & Lucero-Wagoner, B. (2000). The pupillary system. In J. T. Cacioppo, L. G. Tassinary, & G. G. Berntson (Eds.), *Handbook of psychophysiology* (2nd ed., pp. 142–162). Cambridge University Press.

[CR11] Berglund, B., Berglund, U., Ekman, G., & Frankehaeuser, M. (1969). The influence of auditory stimulus intensity on apparent duration. *Scandinavian Journal of Psychology,**10*, 21–26. 10.1111/j.1467-9450.1969.tb00003.x 5353394 10.1111/j.1467-9450.1969.tb00003.x

[CR12] Berridge, C. W., & Waterhouse, B. D. (2003). The locus coeruleus–noradrenergic system: Modulation of behavioral state and state-dependent cognitive processes. *Brain Research Reviews,**42*(1), 33–84. 12668290 10.1016/s0165-0173(03)00143-7

[CR13] Bi, Z., & Zhou, C. (2020). Understanding the computation of time using neural network models. *Proceedings of the National Academy of Sciences,**117*(19), 10530–10540. 10.1073/pnas.1921609117 PMC7229760 32341153

[CR14] Binda, P., Pereverzeva, M., & Murray, S. O. (2013). Pupil constrictions to photographs of the sun. *Journal of Vision,**13*(6), 8–8. 10.1167/13.6.8 23685391

[CR15] Block, R. A., & Gruber, R. P. (2014). Time perception, attention, and memory: A selective review. *Acta Psychologica,**149*, 129–133. 24365036 10.1016/j.actpsy.2013.11.003

[CR16] Bradley, M. M., Miccoli, L., Escrig, M. A., & Lang, P. J. (2008). The pupil as a measure of emotional arousal and autonomic activation. *Psychophysiology,**45*(4), 602–607. 18282202 10.1111/j.1469-8986.2008.00654.x PMC3612940

[CR17] Brigner, W. L. (1986). The effect of stimulus brightness on the perception of time. *Perceptual and Motor Skills,**62*(1), 83–86.

[CR18] Brown, S. W. (1995). Time, change, and motion: The effects of stimulus movement on temporal perception. *Perception & Psychophysics,**57*, 105–116. 10.3758/BF03211853 7885802 10.3758/bf03211853

[CR19] Brown, S. W. (1997). Attentional resources in timing: Interference effects in concurrent temporal and nontemporal working memory tasks. *Perception & Psychophysics,**59*, 1118–1140. 9360484 10.3758/bf03205526

[CR20] Brown, V. A. (2021). An introduction to linear mixed-effects modeling in R. *Advances in Methods and Practices in Psychological Science*, *4*(1). 10.1177/2515245920960351

[CR21] Bueti, D., & Walsh, V. (2009). The parietal cortex and the representation of time, space, number and other magnitudes. *Philosophical Transactions of the Royal Society B: Biological Sciences,**364*(1525), 1831–1840. 10.1098/rstb.2009.0028 PMC2685826 19487186

[CR22] Buhusi, C. V., & Meck, W. H. (2005). What makes us tick? Functional and neural mechanisms of interval timing. *Nature Reviews Neuroscience,**6*(10), 755–765. 16163383 10.1038/nrn1764

[CR23] Cai, Z. G., & Connell, L. (2015). Space–time interdependence: Evidence against asymmetric mapping between time and space. *Cognition,**136*, 268–281. 25506776 10.1016/j.cognition.2014.11.039

[CR24] Calvert, G. A., Spence, C., & Stein, B. E. (Eds.). (2004). *The handbook of multisensory processes.* MIT Press.

[CR25] Cantlon, J. F., Platt, M. L., & Brannon, E. M. (2009). Beyond the number domain. *Trends in Cognitive Sciences,**13*(2), 83–91. 19131268 10.1016/j.tics.2008.11.007 PMC2709421

[CR26] Casasanto, D., & Boroditsky, L. (2008). Time in the mind: Using space to think about time. *Cognition,**106*(2), 579–593. 17509553 10.1016/j.cognition.2007.03.004

[CR27] Casini, L., & Macar, F. (1997). Effects of attention manipulation on judgments of duration and of intensity in the visual modality. *Memory & Cognition,**25*(6), 812–818. 9421567 10.3758/bf03211325

[CR28] Church, R. M. (1984). Properties of the internal clock. *Annals of the New York Academy of Sciences,**423*(1), 566–582. 6588815 10.1111/j.1749-6632.1984.tb23459.x

[CR29] Coull, J. T., Cheng, R. K., & Meck, W. H. (2011). Neuroanatomical and neurochemical substrates of timing. *Neuropsychopharmacology,**36*(1), 3–25. 20668434 10.1038/npp.2010.113 PMC3055517

[CR30] Coull, J. T., Vidal, F., Nazarian, B., & Macar, F. (2004). Functional anatomy of the attentional modulation of time estimation. *Science,**303*(5663), 1506–1508. 15001776 10.1126/science.1091573

[CR31] Creelman, C. D. (1962). Human discrimination of auditory duration. *The Journal of the Acoustical Society of America,**34*(5), 582–593.

[CR32] Dalmaijer, E. S., Mathôt, S., & Van der Stigchel, S. (2014). PyGaze: An open-source, cross-platform toolbox for minimal-effort programming of eyetracking experiments. *Behavior Research Methods,**46*, 913–921. 24258321 10.3758/s13428-013-0422-2

[CR33] de Hevia, M. D., Izard, V., Coubart, A., Spelke, E. S., & Streri, A. (2014). Representations of space, time, and number in neonates. *Proceedings of the National Academy of Sciences,**111*, 4809–4813. 10.1073/pnas.1323628111 PMC3977279 24639511

[CR34] Driver, J., & Noesselt, T. (2008). Multisensory interplay reveals crossmodal influences on ‘sensory-specific’ brain regions, neural responses, and judgments. *Neuron,**57*(1), 11–23. 18184561 10.1016/j.neuron.2007.12.013 PMC2427054

[CR35] Droit-Volet, S. (2013). Time perception, emotions and mood disorders. *Journal of Physiology-Paris,**107*(4), 255–264. 23542546 10.1016/j.jphysparis.2013.03.005

[CR36] Droit-Volet, S., & Meck, W. H. (2007). How emotions colour our perception of time. *Trends in Cognitive Sciences,**11*(12), 504–513. 18023604 10.1016/j.tics.2007.09.008

[CR37] Droit-Volet, S., & Wearden, J. H. (2002). Speeding up an internal clock in children? Effects of visual flicker on subjective duration. *The Quarterly Journal of Experimental Psychology: Section B,**55*(3), 193–211. 10.1080/02724990143000252 12188524

[CR38] Eagleman, D. M. (2008). Human time perception and its illusions. *Current Opinion in Neurobiology,**18*(2), 131–136. 18639634 10.1016/j.conb.2008.06.002 PMC2866156

[CR39] Eagleman, D. M., & Pariyadath, V. (2009). Is subjective duration a signature of coding efficiency? *Philosophical Transactions of the Royal Society B: Biological Sciences,**364*(1525), 1841–1851. 10.1098/rstb.2009.0026 PMC2685825 19487187

[CR40] Einhauser, W., Koch, C., & Carter, O. (2010). Pupil dilation betrays the timing of decisions. *Frontiers in Human Neuroscience, 4,* Article 946. 10.3389/fnhum.2010.00018 PMC2831633 20204145

[CR41] Fortin, C. (2003). Attentional time-sharing in interval timing. In H. Helfrich (Ed.), *Time and mind II: Information-processing perspectives* (pp. 235–260). Hogrefe & Huber Publishers.

[CR42] Fortin, C., & Rousseau, R. (1998). Interference from short-term memory processing on encoding and reproducing brief durations. *Psychological Research Psychologische Forschung,**61*(4), 269–276. 9870294 10.1007/s004260050031

[CR43] Fortin, C., & Rousseau, R. (1987). Time estimation as an index of processing demand in memory search. *Attention, Perception, and Psychophysics,**42*, 377–382. 10.3758/BF03203095 10.3758/bf03203095 3684495

[CR44] Fortin, C., & Massé, N. (2000). Expecting a break in time estimation: Attentional time-sharing without concurrent processing. *Journal of Experimental Psychology: Human Perception and Performance,**26*(6), 1788. 11129374 10.1037//0096-1523.26.6.1788

[CR45] García-Pérez, M. A. (2014a). Adaptive psychophysical methods for nonmonotonic psychometric functions. *Attention, Perception, & Psychophysics,**76*(6), 1646–1666. 10.3758/s13414-013-0574-2 24197504

[CR46] García-Pérez, M. A. (2014b). Does time ever fly or slow down? The difficult interpretation of psychophysical data on time perception. *Frontiers in Human Neuroscience, 8*, Article 415. 10.3389/fnhum.2014.00415 PMC4051264 24959133

[CR47] Gibbon, J. (1977). Scalar expectancy theory and Weber’s law in animal timing. *Psychological Review,**84*(3), 279–325.

[CR48] Gibbon, J., Church, R. M., & Meck, W. H. (1984). Scalar timing in memory. *Annals of the New York Academy of Sciences,**423*(1), 52–77. 6588812 10.1111/j.1749-6632.1984.tb23417.x

[CR49] Gil, S., & Droit-Volet, S. (2012). Emotional time distortions: The fundamental role of arousal. *Cognition & Emotion,**26*(5), 847–862. 22296278 10.1080/02699931.2011.625401

[CR50] Gilzenrat, M. S., Nieuwenhuis, S., Jepma, M., & Cohen, J. D. (2010). Pupil diameter tracks changes in control state predicted by the adaptive gain theory of locus coeruleus function. *Cognitive, Affective, & Behavioral Neuroscience,**10*(2), 252–269. 10.3758/CABN.10.2.252 PMC3403821 20498349

[CR51] Goel, A., & Buonomano, D. V. (2014). Timing as an intrinsic property of neural networks: Evidence from in vivo and in vitro experiments. *Philosophical transactions of the Royal Society B: Biological sciences, 369*(1637), Article 20120460. 10.1098/rstb.2012.0460 PMC3895985 24446494

[CR52] Goldinger, S. D., & Papesh, M. H. (2012). Pupil dilation reflects the creation and retrieval of memories. *Current Directions in Psychological Science,**21*(2), 90–95. 29093614 10.1177/0963721412436811 PMC5662122

[CR53] Goldstone, S., & Goldfarb, J. L. (1964a). Auditory and visual time judgment. *The Journal of General Psychology,**70*(2), 369–387. 14140126 10.1080/00221309.1964.9920609

[CR54] Goldstone, S., & Goldfarb, J. L. (1964b). Brightness and duration in the perception of time. *Perceptual and Motor Skills,**19*(1), 39–42. 10.2466/pms.1964.19.2.606 14214737

[CR55] Goldstone, S., Lhamon, W. T., & Sechzer, J. (1978). Light intensity and judged duration. *Bulletin of the Psychonomic Society,**12*(1), 83–84.

[CR56] Grondin, S. (Ed.). (2008). Methods for studying psychological time. *Psychology of time.* Springer. [Google Books]

[CR57] Grondin, S. (2010). Timing and time perception: A review of recent behavioral and neuroscience findings and theoretical directions. *Attention, Perception, & Psychophysics,**72*(3), 561–582. 10.3758/APP.72.3.561 20348562

[CR58] Grondin, S. (2024). *The Processing of Short Time Intervals: Some Critical Issues*. Springer. 10.1007/978-3-031-60183-5_3 38918345

[CR59] Herbst, S. K., Javadi, A. H., van der Meer, E., & Busch, N. A. (2013). How long depends on how fast—Perceived flicker dilates subjective duration. *PLOS ONE, 8*(10), Article e76074. 10.1371/journal.pone.0076074 PMC3806760 24194829

[CR60] Hurvich, L. M., & Jameson, D. (1966). *The perception of brightness and darkness*. Allyn & Bacon.

[CR61] Ivry, R. B., & Schlerf, J. E. (2008). Dedicated and intrinsic models of time perception. *Trends in Cognitive Sciences,**12*(7), 273–280. 18539519 10.1016/j.tics.2008.04.002 PMC4335014

[CR62] JASP Team. (2023). *JASP* (Version 0.17.3) [Computer software]. https://www.jasp-stats.org

[CR63] Joshi, S., & Gold, J. I. (2020). Pupil size as a window on neural substrates of cognition. *Trends in Cognitive Sciences,**24*(5), 466–480. 32331857 10.1016/j.tics.2020.03.005 PMC7271902

[CR64] Joshi, S., Li, Y., Kalwani, R. M., & Gold, J. I. (2016). Relationships between pupil diameter and neuronal activity in the locus coeruleus, colliculi, and cingulate cortex. *Neuron,**89*(1), 221–234. 26711118 10.1016/j.neuron.2015.11.028 PMC4707070

[CR65] Kanai, R., Paffen, C. L., Hogendoorn, H., & Verstraten, F. A. (2006). Time dilation in dynamic visual display. *Journal of Vision,**6*(12), 8–8. 17209745 10.1167/6.12.8

[CR66] Kaneko, S., & Murakami, I. (2009). Perceived duration of visual motion increases with speed. *Journal of Vision,**9*(7), 14–14. 19761329 10.1167/9.7.14

[CR67] Karsilar, H., & Balcı, F. (2016). Asymmetrical modulation of time perception by increase versus decrease in coherence of motion. *Attention, Perception, & Psychophysics,**78*, 2690–2707. 10.3758/s13414-016-1181-9 27527372

[CR68] Karsilar, H., & Balcı, F. (2019). Symbolism overshadows the effect of physical size in supra-second temporal illusions. *Attention, Perception, & Psychophysics,**81*, 2902–2916. 10.3758/s13414-019-01748-x 31165452

[CR69] Karsilar, H., Kısa, Y. D., & Balcı, F. (2018). Dilation and constriction of subjective time based on observed walking speed. *Frontiers in psychology, 9,* Article 2565. 10.3389/fpsyg.2018.02565 PMC6309241 30627109

[CR70] Karsilar, H., Mathot, S., & Van Rijn, H. (2024). Attention modulates the effects of stimulus brightness and contrast on time perception. *PsyArXiv Preprint*. 10.3758/s13423-026-02893-9 PMC13002725 41857475

[CR71] Kinzuka, Y., Sato, F., Minami, T., & Nakauchi, S. (2021). Effect of glare illusion-induced perceptual brightness on temporal perception. *Psychophysiology,**58*(9), e13851. 34036604 10.1111/psyp.13851 PMC8459261

[CR72] Laeng, B., Sirois, S., & Gredebäck, G. (2012). Pupillometry: A window to the preconscious? *Perspectives on Psychological Science,**7*(1), 18–27. 26168419 10.1177/1745691611427305

[CR73] Lake, J. I., LaBar, K. S., & Meck, W. H. (2016). Emotional modulation of interval timing and time perception. *Neuroscience & Biobehavioral Reviews,**64*, 403–420. 26972824 10.1016/j.neubiorev.2016.03.003 PMC5380120

[CR74] Lejeune, H., & Wearden, J. H. (2009). Vierordt’s *The Experimental Study of the Time Sense* (1868) and its legacy. *European Journal of Cognitive Psychology,**21*(6), 941–960.

[CR75] Lewis, P. A., & Miall, R. C. (2003). Distinct systems for automatic and cognitively controlled time measurement: Evidence from neuroimaging. *Current Opinion in Neurobiology,**13*(2), 250–255. 12744981 10.1016/s0959-4388(03)00036-9

[CR76] Malapani, C., & Fairhurst, S. (2002). Scalar timing in animals and humans. *Learning and Motivation,**33*(1), 156–176.

[CR77] Mathôt, S. (2018). Pupillometry: Psychology, physiology, and function. *Journal of Cognition, 1*(1), Article 16. 10.5334/joc.18 PMC6634360 31517190

[CR78] Mathôt, S., & March, J. (2022). Conducting linguistic experiments online with OpenSesame and OSWeb. *Language Learning*. 10.1111/lang.12509

[CR79] Mathôt, S., Schreij, D., & Theeuwes, J. (2012). OpenSesame: An open-source, graphical experiment builder for the social sciences. *Behavior Research Methods,**44*(2), 314–324. 10.3758/s13428-011-0168-7 22083660 10.3758/s13428-011-0168-7 PMC3356517

[CR80] Mathôt, S., & Van der Stigchel, S. (2015). New light on the mind’s eye: The pupillary light response as active vision. *Current Directions in Psychological Science,**24*(5), 374–378. 26494950 10.1177/0963721415593725 PMC4601080

[CR81] Mathôt, S., & Vilotijević, A. (2023). Methods in cognitive pupillometry: Design, preprocessing, and statistical analysis. *Behavior Research Methods,**55*(6), 3055–3077. 36028608 10.3758/s13428-022-01957-7 PMC10556184

[CR82] Matthews, W. J. (2011). Stimulus repetition and the perception of time: The effects of prior exposure on temporal discrimination, judgment, and production. *PLOS ONE, 6*(5), Article e19815. 10.1371/journal.pone.0019815 PMC3090413 21573020

[CR83] Matthews, W. J., & Meck, W. H. (2016). Temporal cognition: Connecting subjective time to perception, attention, and memory. *Psychological Bulletin,**142*(8), 865–907. 27196725 10.1037/bul0000045

[CR84] Meck, W. H. (2006). Neuroanatomical localization of an internal clock: A functional link between mesolimbic, nigrostriatal, and mesocortical dopaminergic systems. *Brain Research,**1109*(1), 93–107. 16890210 10.1016/j.brainres.2006.06.031

[CR85] Mella, N., Conty, L., & Pouthas, V. (2011). The role of physiological arousal in time perception: Psychophysiological evidence from an emotion regulation paradigm. *Brain and Cognition,**75*(2), 182–187. 21145643 10.1016/j.bandc.2010.11.012

[CR86] Merchant, H., Harrington, D. L., & Meck, W. H. (2013). Neural basis of the perception and estimation of time. *Annual Review of Neuroscience,**36*, 313–336. 10.1146/annurev-neuro-062012-170349 23725000

[CR87] Naber, M., Alvarez, G. A., & Nakayama, K. (2013). Tracking the allocation of attention using human pupillary oscillations. *Frontiers in Psychology, 4,* Article 919. 10.3389/fpsyg.2013.00919 PMC3857913 24368904

[CR88] Nather, F. C., & Bueno, J. L. (2011). Time perception and arousal: Is time perception enhanced, speeded, or both under conditions of arousal? *Psychology & Neuroscience,**4*(2), 255–262.

[CR89] Nather, F. C., Bueno, J. L., Bigand, E., & Droit-Volet, S. (2011). Time changes with the embodiment of another’s body posture. *PLOS ONE, 6*(5), Article e19818. 10.1371/journal.pone.0019818 PMC3103514 21637759

[CR90] Ono, F., & Kawahara, J. I. (2007). The subjective size of visual stimuli affects the perceived duration of their presentation. *Perception & Psychophysics,**69*(6), 952–957. 18018976 10.3758/bf03193932

[CR91] Palmer, S. E. (1999). *Vision science: Photons to phenomenology.* MIT Press.

[CR92] Pariyadath, V., & Eagleman, D. M. (2007). The effect of predictability on subjective duration. *PLOS ONE, 2*(11), Article e1264. 10.1371/journal.pone.0001264 PMC2082074 18043760

[CR93] Partala, T., & Surakka, V. (2003). Pupil size variation as an indication of affective processing. *International Journal of Human-Computer Studies,**59*(1/2), 185–198.

[CR94] Peirce, J. W. (2007). PsychoPy—psychophysics software in Python. *Journal of Neuroscience Methods,**162*(1–2), 8–13. 17254636 10.1016/j.jneumeth.2006.11.017 PMC2018741

[CR95] Penney, T. B., & Cheng, X. (2018). Duration bisection: Q user’s guide. In A. Vatakis, F. Balcı, M. Di Luca, & Á. Correa (Eds.), *Timing and time perception: Procedures, measures, & applications* (pp. 98–127). Brill.

[CR96] Penney, T. B., Gibbon, J., & Meck, W. H. (2000). Differential effects of auditory and visual signals on clock speed and temporal memory. *Journal of Experimental Psychology: Human Perception and Performance,**26*(6), 1770–1787. 11129373 10.1037//0096-1523.26.6.1770

[CR97] Penton-Voak, I. S., Edwards, H., Percival, A., & Wearden, J. H. (1996). Speeding up an internal clock in humans? Effects of click trains on subjective duration. *Journal of Experimental Psychology: Animal Behavior Processes,**22*(3), 307–320. 8691161 10.1037//0097-7403.22.3.307

[CR98] Petrizzo, I., Pellegrino, M., Anobile, G., Doricchi, F., & Arrighi, R. (2023). Top-down determinants of the numerosity–time interaction. *Scientific Reports, 13*(1), Article 21098. 10.1038/s41598-023-47507-9 PMC10689472 38036544

[CR99] Rammsayer, T. H., & Lima, S. D. (1991). Duration discrimination of filled and empty auditory intervals: Cognitive and perceptual factors. *Perception & Psychophysics,**50*(6), 565–574. 1780204 10.3758/bf03207541

[CR100] Rammsayer, T. H., & Verner, M. (2016). Evidence for different processes involved in the effects of nontemporal stimulus size and numerical digit value on duration judgments. *Journal of Vision,**16*(7), 13–13. 10.1167/16.7.13 PMC4900137 27191941

[CR101] Raphan, T., Dorokhin, E., & Delamater, A. R. (2019). Modeling interval timing by recurrent neural nets. *Frontiers in Integrative Neuroscience, 13,* Article 46. 10.3389/fnint.2019.00046 PMC6724642 31555104

[CR102] Rohenkohl, G., Cravo, A. M., Wyart, V., & Nobre, A. C. (2012). Temporal expectation improves the quality of sensory information. *Journal of Neuroscience,**32*(24), 8424–8428. 22699922 10.1523/JNEUROSCI.0804-12.2012 PMC4235252

[CR103] RStudio Team. (2020). *RStudio: Integrated development for R* [Computer software]. RStudio. http://www.rstudio.com/

[CR104] Sackett, A. M., Meyvis, T., Nelson, L. D., Converse, B. A., & Sackett, A. L. (2010). You’re having fun when time flies: The hedonic consequences of subjective time progression. *Psychological Science,**21*(1), 111–117. 20424031 10.1177/0956797609354832

[CR105] Salet, J. M., Kruijne, W., van Rijn, H., Los, S. A., & Meeter, M. (2022). FMTP: A unifying computational framework of temporal preparation across time scales. *Psychological Review,**129*(5), 911. 35420847 10.1037/rev0000356

[CR106] Smith, S. D., McIver, T. A., Di Nella, M. S., & Crease, M. L. (2011). The effects of valence and arousal on the emotional modulation of time perception: Evidence for multiple stages of processing. *Emotion, 11*(6), Article 1305. 10.1037/a0026145 22142208

[CR107] Staddon, J., & Higa, J. (1999). Time and memory: Towards a pacemaker-free theory of interval timing. *Journal of the Experimental Analysis of Behavior,**71*, 215–251. 10.1901/jeab.1999.71-215 10220931 10.1901/jeab.1999.71-215 PMC1284701

[CR108] Stein, B. E., & Meredith, M. A. (1993). *The merging of the senses*. MIT Press.

[CR109] Stein, B. E., Stanford, T. R., & Rowland, B. A. (2009). The neural basis of multisensory integration in the midbrain: Its organization and maturation. *Hearing Research,**258*(1/2), 4–15. 19345256 10.1016/j.heares.2009.03.012 PMC2787841

[CR110] Stone, J. V. (2014). Using reaction times and binary responses to estimate psychophysical performance: An information-theoretic analysis. *Frontiers in Neuroscience, 8*, Article 35. 10.3389/fnins.2014.00035 PMC3941087 24624053

[CR111] Sulutvedt, U., Zavagno, D., Lubell, J., Leknes, S., de Rodez Benavent, S. A., & Laeng, B. (2021). Brightness perception changes related to pupil size. *Vision Research,**178*, 41–47. 33113435 10.1016/j.visres.2020.09.004

[CR112] Suzuki, M., Kunimatsu, J., & Tanaka, M. (2016). Correlation between pupil size and subjective passage of time in non-human primates. *Journal of Neuroscience,**36*(44), 11331–11337. 27807173 10.1523/JNEUROSCI.2533-16.2016 PMC6601963

[CR113] Thomas, E. A., & Cantor, N. E. (1976). Simultaneous time and size perception. *Perception & Psychophysics,**19*(4), 353–360.

[CR114] Treisman, M. (1963). Temporal discrimination and the indifference interval: Implications for a model of the “internal clock.” *Psychological Monographs: General and Applied,**77*(13), 1–31. 10.1037/h0093864 5877542

[CR115] Treisman, M., Faulkner, A., Naish, P. L., & Brogan, D. (1990). The internal clock: Evidence for a temporal oscillator underlying time perception with some estimates of its characteristic frequency. *Perception,**19*(6), 705–743. 2130371 10.1068/p190705

[CR116] Tse, P. U., Intriligator, J., Rivest, J., & Cavanagh, P. (2004). Attention and the subjective expansion of time. *Perception & Psychophysics,**66*(7), 1171–1189. 15751474 10.3758/bf03196844

[CR117] Ulrich, R., Nitschke, J., & Rammsayer, T. (2006). Perceived duration of expected and unexpected stimuli. *Psychological Research Psychologische Forschung,**70*(2), 77–87. 15609031 10.1007/s00426-004-0195-4

[CR118] van Rijn, H., Dalenberg, J. R., Borst, J. P., & Sprenger, S. A. (2012). Pupil dilation co-varies with memory strength of individual traces in a delayed response paired-associate task. *PLOS ONE, 7*(12), Article e51134. 10.1371/journal.pone.0051134 PMC3515525 23227244

[CR119] van Rijn, H., Gu, B. M., & Meck, W. H. (2014). Dedicated clock/timing-circuit theories of time perception and timed performance. In H. Merchant & V. de Lafuente (Eds.), *Neurobiology of interval timing* (pp. 75–99). Springer. 10.1007/978-1-4939-1782-2_5 25358706

[CR120] Van Volkinburg, H., & Balsam, P. (2014). Effects of emotional valence and arousal on time perception. *Timing & Time Perception,**2*(3), 360–378. 27110491 10.1163/22134468-00002034 PMC4838289

[CR121] van Wassenhove, V. (2009). Minding time in an amodal representational space. *Philosophical Transactions of the Royal Society B: Biological Sciences,**364*(1525), 1815–1830. 10.1098/rstb.2009.0023 PMC2685822 19487185

[CR122] van Wassenhove, V., Buonomano, D. V., Shimojo, S., & Shams, L. (2008). Distortions of subjective time perception within and across senses. *PLOS ONE, 3*(1), Article e1437. 10.1371/journal.pone.0001437 PMC2174530 18197248

[CR123] Vidaud-Laperrière, K., Brunel, L., Syssau-Vaccarella, A., & Charras, P. (2022). Exploring spatiotemporal interactions: On the superiority of time over space. *Attention, Perception, & Psychophysics,**84*(8), 2582–2595. 10.3758/s13414-022-02546-8 36229633

[CR124] Vidotto, G., Anselmi, P., & Robusto, E. (2019). New perspectives in computing the point of subjective equality using Rasch models. *Frontiers in Psychology, 10*, Article 2793. 10.3389/fpsyg.2019.02793 PMC6927926 31920838

[CR125] Walsh, V. (2003). A theory of magnitude: Common cortical metrics of time, space and quantity. *Trends in Cognitive Sciences,**7*(11), 483–488. 14585444 10.1016/j.tics.2003.09.002

[CR126] Wang, P., & Reynaud, A. (2023). The random step method for measuring the point of subjective equality. *Vision,**7*(4), 74. 37987294 10.3390/vision7040074 PMC10661322

[CR127] Warda, S., Simola, J., & Terhune, D. B. (2022). Pupillometry tracks errors in interval timing. *Behavioral Neuroscience,**136*(5), 495–502. 36222640 10.1037/bne0000533 PMC9552500

[CR128] Wearden, J. H. (1991). Human performance on an analogue of an interval bisection task. *The Quarterly Journal of Experimental Psychology,**43*(1), 59–81. 2017575

[CR129] Wearden, J. H. (1999). “Beyond the fields we know...”: Exploring and developing scalar timing theory. *Behavioural Processes, 45*(1/3), 3–21. 10.1016/s0376-6357(99)00006-6 24897524

[CR130] Wearden, J. H. (2003). Applying the scalar timing model to human time psychology: Progress and challenges. In H. Helfrich (Ed.), *Time and mind II: Information processing perspectives* (pp. 21–39). Hogrefe & Huber Publishers.

[CR131] Wearden, J. H. (2008). Slowing down an internal clock: Implications for accounts of performance on four timing tasks. *The Quarterly Journal of Experimental Psychology,**61*(2), 263–274. 17853194 10.1080/17470210601154610

[CR132] Wearden, J. H., & Ferrara, A. (1995). Stimulus range effects in temporal bisection by humans. *The Quarterly Journal of Experimental Psychology,**48*(2), 289–310. 8532899

[CR133] Wearden, J. H., Edwards, H., Fakhri, M., & Percival, A. (1998). Why “sounds are judged longer than lights”: Application of a model of the internal clock in humans. *The Quarterly Journal of Experimental Psychology: Section B,**51*(2), 97–120. 10.1080/713932672 9621837

[CR134] Wittmann, M. (2013). The inner sense of time: How the brain creates a representation of duration. *Nature Reviews Neuroscience,**14*(3), 217–223. 23403747 10.1038/nrn3452

[CR135] Wittmann, M., van Wassenhove, V., Craig, A. D., & Paulus, M. P. (2010). The neural substrates of subjective time dilation. *Frontiers in Human Neuroscience,**4*, 2. 20161994 10.3389/neuro.09.002.2010 PMC2820380

[CR136] Xuan, B., Zhang, D., He, S., & Chen, X. (2007). Larger stimuli are judged to last longer. *Journal of Vision,**7*(10), 2–2. 10.1167/7.10.2 17997671

[CR137] Zakay, D. (1998). Attention allocation policy influences prospective timing. *Psychonomic Bulletin & Review,**5*(1), 114–118.

[CR138] Zakay, D., & Block, R. A. (1996). The role of attention in time estimation processes. In M. A. Pastor & J. Artieda (Eds.), *Time, internal clocks and movement* (pp. 143–164). Elsevier.

[CR139] Zakay, D., & Block, R. A. (1997). Temporal cognition. *Current Directions in Psychological Science,**6*(1), 12–16.

